# Validation of use of the miniPCR thermocycler for Ebola and Zika virus detection

**DOI:** 10.1371/journal.pone.0215642

**Published:** 2019-05-09

**Authors:** Everardo González-González, Jackelin Lizeth Mendoza-Ramos, Sara Cristina Pedroza, Aimé Alexandra Cuellar-Monterrubio, Alan Roberto Márquez-Ipiña, Daniel Lira-Serhan, Grissel Trujillo-de Santiago, Mario Moisés Alvarez

**Affiliations:** 1 Centro de Biotecnología-FEMSA, Tecnologico de Monterrey, CP, Monterrey, Nuevo León, México; 2 Departamento de Bioingeniería, Tecnologico de Monterrey, CP, Monterrey, Nuevo León, México; 3 Departamento de Ingeniería Mecátrónica y Eléctrica, Tecnologico de Monterrey, CP, Monterrey, Nuevo León, México; Mercy Hospital, SIERRA LEONE

## Abstract

The development of point-of-care (POC) diagnostic systems has received well-deserved attention in recent years in the scientific literature, and many experimental systems show great promise in real settings. However, in the case of an epidemic emergency (or a natural disaster), the first line of response should be based on commercially available and validated resources. Here, we compare the performance and ease of use of the miniPCR, a recently commercially available compact and portable PCR device, and a conventional thermocycler for the diagnostics of viral nucleic acids. We used both thermocyclers to detect and amplify Ebola and Zika DNA sequences of different lengths (in the range of 91 to 300 nucleotides) at different concentrations (in the range of ~50 to 4.0 x 10^8^ DNA copies). Our results suggest that the performance of both thermocyclers is quite similar. Moreover, the portability, ease of use, and reproducibility of the miniPCR makes it a reliable alternative for point-of-care nucleic acid detection and amplification.

## Introduction

The development of cost-efficient diagnostic point of care (POC) systems for the opportune diagnosis of infectious diseases is a research niche of high relevance [1,2]. The recent pandemic/epidemic episodes associated with viral diseases (e.g., influenza epi-centered in México in 2009 [3,4], Ebola in West Africa in 2013–2015 [5–7], and Zika in Latin America and Southeast Asia in 2016 [8–10]) are tangible and cruel reminders of the need for portable, low-cost, and easy-to-use diagnostic systems that can effectively address epidemic episodes in remote or underprivileged areas [5,9,11,12].

Many methodologies have been proposed to deliver cost/effective diagnosis (i.e., those based on immunoassays or specific gene hybridization [7,13–16]); however, nucleic acid amplification continues to be the gold standard for the detection of viral diseases in early stages [9,11,17–19]. Among these, the polymerase chain reaction (PCR) is the most established method for molecular diagnostics [10,20]. However, bringing the benefits of PCR to remote or unprivileged areas has been challenging due to the need for centralized laboratory settings to accomplish traditional PCR testing [21–23], coupled with the still inherently high cost of traditional PCR equipment and reagents [24]. To resolve this drawback, multiple studies have proposed and validated the use of compact PCR-based methods and devices for POC settings [22,25,26].

The first wave of miniaturized PCR machines has only recently become commercially available [27]. The miniPCR from Amplyus (MA, USA) is one of the first highly compact PCR units on the international market [28]. The first miniPCR units reached the marketplace in 2015, with an approximate cost of $600 USD (versus $3000 USD for a conventional PCR thermocycler) [27]. Thus far, only a few papers have been published that directly relate to the validation of use of the miniPCR system as a diagnostic tool[29–33]. For example, Guevara et al. demonstrated its use on the study of wild populations of lemurs in the field [29]. Similarly, Pomerantz et al. demonstrated the combined used of miniPCR and minIon sequencer (a miniaturized sequencer) for the rapid identification of species in the Ecuadorian rainforest [30] (i.e., less than 24 hours after data collection). Boguraev et al. successfully amplified zebrafish DNA in a remarkably non-conventional setting—aboard the International Space Station—and they were able to amplify bisulfite-treated DNA to determine epigenetic variations. They found that methylation-specific primers differentially amplified bisulfite-treated DNA just as would be expected under standard laboratory conditions on Earth [31]. Recently, Zaky et al. used the miniPCR system for the detection of Brugia parasites in mosquitos [33]. The commercial availability, low price (as compared to conventional thermocyclers), portability, and user friendliness make the miniPCR an attractive and tangible solution that effectively brings PCR analysis to the POC.

In the case of evaluating the spread of an infectious disease, the deployment of any diagnostic effort has to rely first on commercially available equipment [34]. Consequently, a comparison of current commercially available compact PCR platforms is of paramount importance. Here, we compare the sensitivity, reproducibility, and convenience of use of the miniPCR (www.minipcr.com) versus a conventional and commercially available PCR thermocycler for the detection and amplification of synthetic samples of Ebola virus and Zika (ZIKV) virus. The *Zaire ebolavirus* (EBOV)[35] and ZIKV have caused the two most recent widespread international epidemic emergencies. From 2014 to 2016, West Africa was the site of the most important Ebola epidemic documented so far; more than 20,000 persons were infected and more than 11,000 died of this disease. From 2015 to 2016, a ZIKV epidemic episode affecting Las Americas reached alarming proportions. The association of ZIKV with recent cases of microcephalic births has increased the concern about establishing systems for early diagnosis [9,36].

## Materials and methods

### Equipment specifications

We ran equivalent sets of amplification experiments in a commercial standard thermocycler (MaxyGene from Axygen, CA, USA) and a miniPCR from Amplyus (MA, USA). The MaxyGene unit can run 96 amplifications simultaneously, has dimensions of 25×25×25 cm, and a weight of 3.5 kg. This unit requires 120V (AC) and 3.5 A to operate. The miniPCR can run 8 amplifications in parallel, has dimensions of 20×5×15 cm, and a weight of 0.7 kg. This unit requires 120V (AC) and 3.5 A to operate.

**Fig 1:**
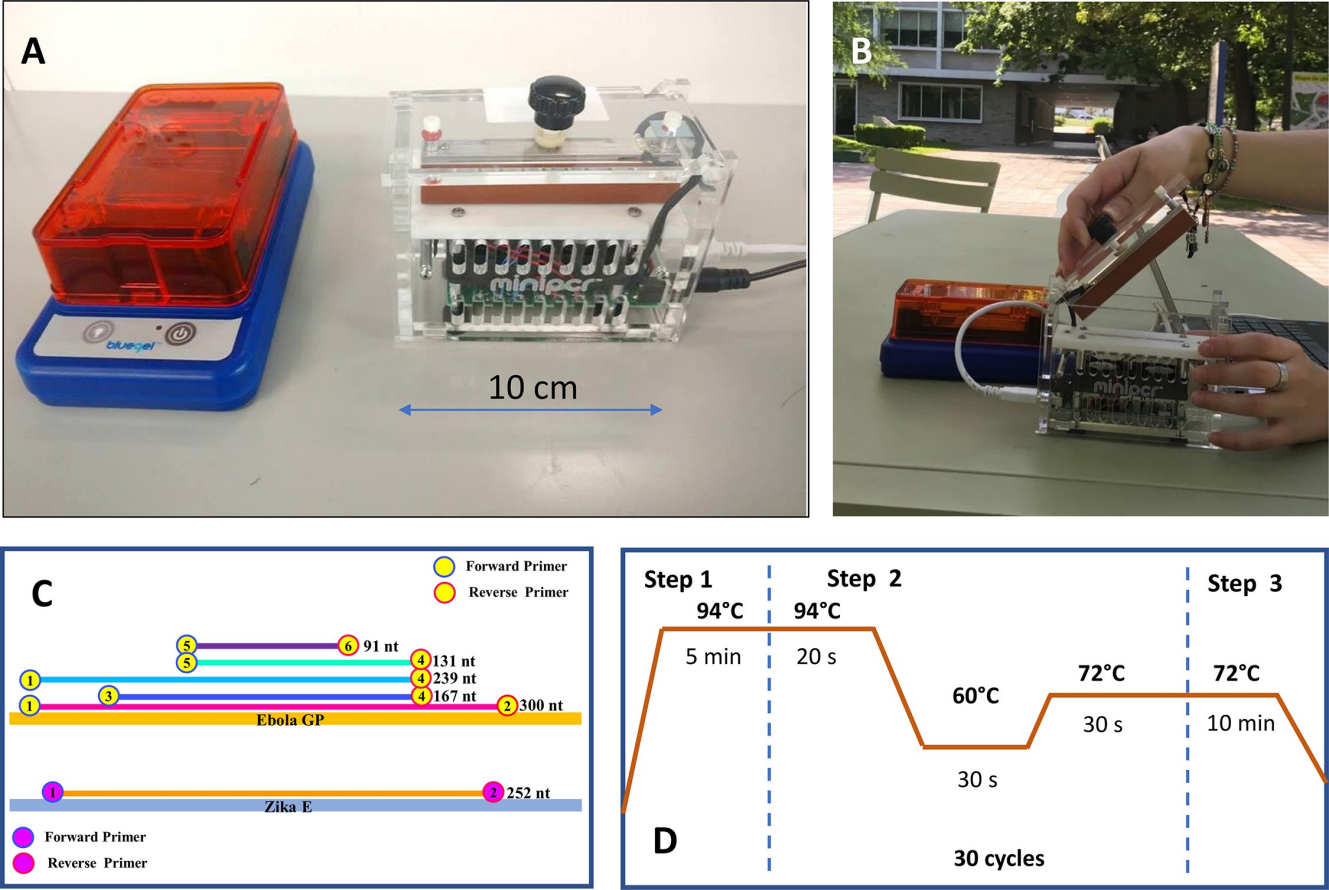
A) The miniPCR thermocycler and blueGel electrophoretic chamber. B) Outdoor use of the miniPCR device. Portability, ease of use, and low toxicity of the reagents allow genetic material amplification outside the laboratory. C) Amplicons of various lengths can be obtained using different sets of primers targeting a sequence encoding the Ebola virus GP protein. D) Temperature cycling scheme used in our PCR protocol.

A commercial power supply (PowerPac from Bio-Rad, CA, USA) was used to operate the electrophoresis unit in which the agarose gels were run to reveal the amplification products obtained by both the MaxyGene and the miniPCR thermocyclers. A Bio-Rad ChemiDoc XRS imaging system was used for end-point PCR detection. Alternatively, the miniPCR unit has its own blueGel electrophoresis unit (Fig 1A and 1B), powered by 120 AC volts, and photo-documentation can be done using a smartphone camera.

### Samples and sample preparation

We used synthetic samples containing EBOV and ZIKV nucleic acids in our validation experiments. We prepared the EBOV samples by cloning a genetic sequence (Table 1) encoding the production of the EBOV GP capsid protein in an *Escherichia* coli strain (TOP10). Similarly, we cloned a genetic sequence encoding the production of the ZIKV E capsid protein in an *E*. *coli* strain (TOP10) to produce ZIKV nucleic acid material (Table 1). The EBOV sequence was designed based on the consensus of GP sequences of the *Zaire ebolavirus* documented in GenBank from 1976–2014 (S1 Fig). The ZIKV sequence was designed by considering the consensus of the E gene sequences of the ZIKV strains documented globally in GenBank from 2013–2016 (S2 Fig).

**Table 1:** Nucleotide sequences used to engineer bacterial strains to produce Ebola virus (EBOV) and Zika virus (ZIKV) nucleic acids.

Target gene	Nucleotide sequence
Ebola GP^α^ protein (capsid)	**EBOV-GP:** ATGTCTACCGAGTCTATGATTAGGGACGTGGAACTGGCTGAGGAGGCACTGCCCAAAAAAACCGGCGGACCACAGGGCTCTAGGAGATGTCTGTTTCTGTCTCTGTTCTCTTTTCTCATCGTGGCTGGCGCTACAACACTCTTCTGTCTGCTCCATTTCGGCGTGATTGGACCACAGCGAGAGGAATTTCCCCGGGATCTGTCACTCATTTCACCACTGGCACAGGCTGTCCGATCTTCATCTCGGACTCCATCCGACAAACCTGTCGCCCATGTCGTCGCCAACCCACAGGCCGAGGGCCAGCTCCAGTGGCTCAATAGGAGGGCAAACGCTCTGCTCGCCAATGGCGTGGAACTCCGGGATAACCAGCTCGTCGTGCCTAGTGAGGGACTGTACCTCATCTACTCCCAGGTGCTGTTTAAGGGCCAGGGATGTCCTTCTACACATGTGCTGCTCACACACACAATTTCACGGATCGCCGTGTCTTACCAGACTAAAGTCAATCTGCTCTCTGCCATCAAATCCCCATGTCAGCGGGAAACACCTGAGGGCGCTGAGGCTAAACCTTGGTACGAACCCATCTACCTCGGAGGCGTGTTCCAGCTGGAGAAGGGCGATAGACTGAGTGCTGAGATCAATCGACCCGACTACCTCGACTTTGCCGAATCTGGCCAGGTCTACTTTGGCATCATTGCTCTGGGCCTCGAGGGCCGAGCTCATGGCGCACCTAGGCCTTTGAATTCCTCTACCGAGTCTATGATTAGGGACGTGGAACTGGCTGAGGAGGCACTGCCCAAAAAAACCGGCGGACCACAGGGCTCTAGGAGATGTCTGTTTCTGTCTCTGTTCTCTTTTCTCATCGTGGCTGGCGCTACAACACTCTTCTGTCTGCTCCATTTCGGCGTGATTGGACCACAGCGAGAGGAATTTCCCCGGGATCTGTCACTCATTTCACCACTGGCACAGGCTGTCCGATCTTCATCTCGGACTCCATCCGACAAACCTGTCGCCCATGTCGTCGCCAACCCACAGGCCGAGGGCCAGCTCCAGTGGCTCAATAGGAGGGCAAACGCTCTGCTCGCCAATGGCGTGGAACTCCGGGATAACCAGCTCGTCGTGCCTAGTGAGGGACTGTACCTCATCTACTCCCAGGTGCTGTTTAAGGGCCAGGGATGTCCTTCTACACATGTGCTGCTCACACACACAATTTCACGGATCGCCGTGTCTTACCAGACTAAAGTCAATCTGCTCTCTGCCATCAAATCCCCATGTCAGCGGGAAACACCTGAGGGCGCTGAGGCTAAACCTTGGTACGAACCCATCTACCTCGGAGGCGTGTTCCAGCTGGAGAAGGGCGATAGACTGAGTGCTGAGATCAATCGACCCGACTACCTCGACTTTGCCGAATCTGGCCAGGTCTACTTTGGCATCATTGCTCTGGGCCTCGAGGGCCGAGCTCATGGCGCACCTAGGCCTTTGAATTCCTCTACCGAGTCTATGATTAGGGACGTGGAACTGGCTGAGGAGGCACTGCCCAAAAAAACCGGCGGACCACAGGGCTCTAGGAGATGTCTGTTTCTGTCTCTGTTCTCTTTTCTCATCGTGGCTGGCGCTACAACACTCTTCTGTCTGCTCCATTTCGGCGTGATTGGACCACAGCGAGAGGAATTTCCCCGGGATCTGTCACTCATTTCACCACTGGCACAGGCTGTCCGATCTTCATCTCGGACTCCATCCGACAAACCTGTCGCCCATGTCGTCGCCAACCCACAGGCCGAGGGCCAGCTCCAGTGGCTCAATAGGAGGGCAAACGCTCTGCTCGCCAATGGCGTGGAACTCCGGGATAACCAGCTCGTCGTGCCTAGTGAGGGACTGTACCTCATCTACTCCCAGGTGCTGTTTAAGGGCCAGGGATGTCCTTCTACACATGTGCTGCTCACACACACAATTTCACGGATCGCCGTGTCTTACCAGACTAAAGTCAATCTGCTCTCTGCCATCAAATCCCCATGTCAGCGGGAAACACCTGAGGGCGCTGAGGCTAAACCTTGGTACGAACCCATCTACCTCGGAGGCGTGTTCCAGCTGGAGAAGGGCGATAGACTGAGTGCTGAGATCAATCGACCCGACTACCTCGACTTTGCCGAATCTGGCCAGGTCTACTTTGGCATCATTGCTCTGGGCCTCGAGGGCCGAGCTCATGGCGCACCTAGGCCTTTGA
Zika E^β^ protein (capside)	**ZIKV-E:** ATGATCAGGTGCATAGGAGTCAGCAATAGGGACTTTGTGGAAGGTATGTCAGGTGGGACTTGGGTTGATGTTGTCTTGGAACATGGAGGTTGTGTCACCGTAATGGCACAGGACAAACCGACTGTCGACATAGAGCTGGTTACAACAACAGTCAGCAACATGGCGGAGGTAAGATCCTACTGCTATGAGGCATCAATATCAGACATGGCTTCGGACAGCCGCTGCCCAACACAAGGTGAAGCCTACCTTGACAAGCAATCAGACACTCAATATGTCTGCAAAAGAACGTTAGTGGACAGAGGCTGGGGAAATGGATGTGGACTTTTTGGCAAAGGGAGCCTGGTGACATGCGCTAAGTTTGCATGCTCCAAGAAAATGACCGGGAAGAGCATCCAGCCAGAGAATCTGGAGTACCGGATAATGCTGTCAGTTCATGGCTCCCAGCACAGTGGGATGATCGTTAATGACACAGGACATGAAACTGATGAGAATAGAGCGAAGGTTGAGATAACGCCCAATTCACCAAGAGCCGAAGCCACCCTGGGGGGTTTTGGAAGCCTAGGACTTGATTGTGAACCGAGGACAGGCCTTGACTTTTCAGATTTGTATTACTTGACTATGAATAACAAGCACTGGTTGGTTCACAAGGAGTGGTTCCACGACATTCCATTACCTTGGCACGCTGGGGCAGACACCGGAACTCCACACTGGAACAACAAAGAAGCACTGGTAGAGTTCAAGGACGCACATGCCAAAAGGCAAACTGTCGTGGTTCTAGGGAGTCAAGAAGGAGCAGTTCACACGGCCCTTGCTGGAGCTCTGGAGGCTGAGATGGATGGTGCAAAGGGAAGGCTGTCCTCTGGCCACTTGAAATGTCGCCTGAAAATGGATAAACTTAGATTGAAGGGCGTGTCATACTCCTTGTGTACCGCAGCGTTCACATTCACCAAGATCCCGGCTGAAACACTGCACGGGACAGTCACAGTGGAGGTACAGTACGCAGGGACAGATGGACCTTGCAAGGTTCCAGCTCAGATGGCGGTGGACATGCAAACTCTGACCCCAGTTGGGAGGTTGATAACCGCTAACCCCGTAATCACTGAAAGCACTGAGAACTCTAAGATGATGCTGGAACTTGATCCACCATTTGGGGACTCTTACATTGTCATAGGAGTCGGGGAGAAGAAGATCACCCACCACTGGCACAGGAGTGGCAGCACCATTGGAAAAGCATTTGAAGCCACTGTGAGAGGTGCCAAGAGAATGGCAGTCTTGGGAGACACAGCCTGGGACTTTGGATCAGTTGGAGGCGCTCTCAACTCATTGGGCAAGGGCATCCATCAAATTTTTGGAGCAGCTTTCAAATCATTGTTTGGAGGAATGTCCTGGTTCTCACAAATTCTCATTGGAACGTTGCTGATGTGGTTGGGTCTGAACACAAAGAATGGATCTATTTCCCTTATGTGCTTGGCCTTAGGGGGAGTGTTGATCTTCTTATCCACAGCCGTCTCTGCTCATCATCATCATCATCATTGA

^α^Most abundant EBOV capsid protein; molecular weight = 74 kDa

^β^Most antigenic ZIKV protein; molecular weight = 54 kDa.

The engineered bacterial strains were cultured overnight in LB-media (250ml) at 37°C in Erlenmeyer flasks under orbital agitation at 200 RPM. After 12 hours of culture, the bacteria were harvested by centrifugation and then lysed using a continuous homogenizer. Synthetic viral DNA was purified from the bacterial samples using the PureYield Plasmid Maxiprep System (Promega, WI, USA). Samples containing different concentrations of synthetic nucleic acids of EBOV and ZIKV were prepared by successive dilutions from stocks containing 400 ng/L of viral nucleic acids.

### Amplification mix

We used REDTaq Ready Mix from Sigma-Aldrich (USA), and followed the recommended protocol: 5 μL Readymix, 1 μL of forward primer, 1 μL of reverse primer (for a final concentration of 0.5 μM), 1 μL of DNA template (3ng to 5 x 10^−7^ ng; ~ 66 to 3.9 x 10^8^ DNA copies), and nuclease free water to final volume of reaction 10 μL.

### Primers used

Three different sets of primers were used to target a region of the EBOV GP sequence. One set of primers was used to target a region that encodes the ZIKV E protein (Fig 1C). Sequences of all these primers and their corresponding amplicons are presented in Tables 2 and 3.

**Table 2:** Primer sequences used in PCR amplification experiments and their corresponding amplicons.

Target gene	Primers sequences
Ebola GP gene (capsid)	EBOV1: ACACTACTGGGAAGCTGATCTGGAAAGTCA EBOV2: AGGTGGTGAGGTGCGACACGGCCGCTTCGC EBOV3: AAACCAAGAAGAACCTGACCAGAAAGATCC EBOV4: GCTGTTTTCGCTAGCCATAATCTTATGATC EBOV5: AGGAGCTGTCCTTCACCGTGGTGTCAAATG EBOV6: GTGTTAGTACCCGGGTCGGATGAGGTGCGC
Zika E gene (capsid)	ZIKV1: AGCCGAAGCCACCCTGGGGGGTTTTGGAAG ZIKV2: CCTAGAACCACGACAGTTTGCCTTTTGGCA

**Table 3:** Amplicon sequences generated (and their corresponding length) by each of the primer pairs used in the PCR amplification experiments (see also Fig 1C).

Primers	Amplicon sequence	Amplicon Length (nt)
EBOV1–EBOV2	ACACTACTGGGAAGCTGATCTGGAAAGTCAACCCGGAGATCGACACTACGATCGGAGAGTGGGCCTTTTGGGAAACCAAGAAGAACCTGACCAGAAAGATCCGGTCCGAGGAGCTGTCCTTCACCGTGGTGTCAAATGGCGCCAAGAACATCTCGGGACAGTCTCCCGCGCGCACCTCATCCGACCCGGGTACTAACACCACTACCGAGGATCATAAGATTATGGCTAGCGAAAACAGCTCGGCCATGGTGCAAGTCCACAGCCAGGGACGCGAAGCGGCCGTGTCGCACCTCACCACCT	300
EBOV3–EBOV4	AAACCAAGAAGAACCTGACCAGAAAGATCCGGTCCGAGGAGCTGTCCTTCACCGTGGTGTCAAATGGCGCCAAGAACATCTCGGGACAGTCTCCCGCGCGCACCTCATCCGACCCGGGTACTAACACCACTACCGAGGATCATAAGATTATGGCTAGCGAAAACAGC	167
EBOV5–EBOV6	AGGAGCTGTCCTTCACCGTGGTGTCAAATGGCGCCAAGAACATCTCGGGACAGTCTCCCGCGCGCACCTCATCCGACCCGGGTACTAACAC	91
EBOV1–EBOV4	ACACTACTGGGAAGCTGATCTGGAAAGTCAACCCGGAGATCGACACTACGATCGGAGAGTGGGCCTTTTGGGAAACCAAGAAGAACCTGACCAGAAAGATCCGGTCCGAGGAGCTGTCCTTCACCGTGGTGTCAAATGGCGCCAAGAACATCTCGGGACAGTCTCCCGCGCGCACCTCATCCGACCCGGGTACTAACACCACTACCGAGGATCATAAGATTATGGCTAGCGAAAACAGC	239
EBOV5–EBOV4	AGGAGCTGTCCTTCACCGTGGTGTCAAATGGCGCCAAGAACATCTCGGGACAGTCTCCCGCGCGCACCTCATCCGACCCGGGTACTAACACCACTACCGAGGATCATAAGATTATGGCTAGCGAAAACAGC	131
ZIKV1–ZIKV2	AGCCGAAGCCACCCTGGGGGGTTTTGGAAGCCTAGGACTTGATTGTGAACCGAGGACAGGCCTTGACTTTTCAGATTTGTATTACTTGACTATGAATAACAAGCACTGGTTGGTTCACAAGGAGTGGTTCCACGACATTCCATTACCTTGGCACGCTGGGGCAGACACCGGAACTCCACACTGGAACAACAAAGAAGCACTGGTAGAGTTCAAGGACGCACATGCCAAAAGGCAAACTGTCGTGGTTCTAGG	252

### Amplification protocols

For all PCR experiments, we used the same three-stage protocol (see Fig 1D) consisting of a denaturation stage at 94°C for 5 min, followed by 30 cycles of 94°C for 20s, 60°C for 30s, and 72°C for 30s, and then a final stage at 72°C for 10 min, with a total duration of 76 and 80 minutes in the MaxyGene and the miniPCR thermocyclers, respectively.

### Documentation of PCR products

We analyzed 10μL of each PCR product using 1% agarose electrophoresis in TAE (Tris-Acetic Acid-EDTA) buffer (Sigma-Aldrich, MO, USA). Gels were dyed with GelGreen (CA, USA) using a 1:10,000 dilution and a current of 110 V supplied by a Bio-Rad PowerPac HC power supply (Bio-Rad, CA, USA) for 50 minutes. We used the Quick-Load Purple 2-Log DNA Ladder (NEB, MA, USA) as molecular a molecular weight marker. We analyzed the gels by UV transillumination using a Bio-Rad ChemiDoc XRS imaging system.

In addition, in some of our experiments, we used the blueGel unit, a portable electrophoresis unit sold by MiniPCR from Amplyus (MA, USA). This is a compact electrophoresis unit (23 × 10 × 7 cm) that weighs 350 g. In these experiments, we analyzed 10μL PCR product using 1% agarose electrophoresis tris-borate-EDTA buffer (TBE). Gels were dyed with GelGreen (CA, USA) using a 1:10,000 dilution, and a current of 48V was supplied by the bluegel built-in power supply (AC 100-240V, 50-60hz).

### Additional quantification strategies for PCR products

We evaluated the total amount of amplified nucleic acids (as quantified by absorbance analysis at 260 nm) and their purity (as quantified by the 260/280 ratio) using a Nanodrop 1000 spectrophotometer (Thermo Fisher Scientific, MA, USA). In these experiments, 10μL of PCR product was purified using the Wizard SV Gel and PCR Clean-Up kits (Promega, WI, USA), and the purified DNA (average yield of 41.5ng/μL) was re-suspended and dissolved in 50 μL of nuclease-free water.

In an additional set of experiments, the amount of amplified DNA was determined by staining with Brilliant III Ultra-Fast SYBR Green QPCR Master Mix (Agilent Technologies CA, USA). We used 0.5 ng of synthetic EBOV or ZIKV as a DNA template, 2 μl of forward primer solution, 2 μl of reverse primer solution, 10 μl of 2× SYBR Green QPCR master mix, and nuclease-free PCR-grade water to adjust the final volume to 20 μl. After the PCR reaction was completed (3 minutes at 95°C; 40 cycles of 5 seconds at 95°C, and 12 seconds at 60°C), the amplification product was transferred to a 7500 Fast Real-Time PCR System (Thermo Fisher Scientific, MA, USA). The amount of amplification product was determined by fluorescence measurements using a SYBR Green filter. For this purpose, 10 cycles of 10 seconds at 21°C were programmed in the Real-Time PCR System. The reading from the fourth cycle was used for statistical analysis.

## Results and discussion

Previous epidemic episodes (for example, those related to EBOV in West Africa in 2014–2016) have proven that an actual emergency does not provide the required timeframe for testing new strategies. This is true for new therapies as well as for diagnosis. In the case of epidemic emergencies (or natural disasters), the first line of response must be based on commercially available and validated resources. Here, we compare the performance of a commercially available portable PCR unit (aimed at several markets, including POC applications) versus that of a conventional, regular-sized PCR thermocycler. The comparison was made in terms of the ability of both units to identify and amplify different synthetically designed genetic sequences of EVOB and ZIKV under the same set of experimental protocols and using the same materials and reagents (see Materials and Methods).

### Engineering of target sequences and primers

For the case of EBOV detection, we designed and synthetized a construct to enable the expression of the EBOV GP protein in *E*.*coli*. GP, an antigenic glycoprotein abundantly present in the EBOV capsid, has been widely studied in literature [6]. The GP construct was cloned in the TOP10 *E*. *coli* strain. The culture and harvest of expressing clones of the bacteria allowed the collection of genetic DNA of EBOV without resorting to the use or propagation of the real virus [4]. For the case of ZIKV detection, we used the same strategy for synthetic production of nucleic acid sequences encoding the ZIKV E protein (Table 1). The flavivirus envelope (E) glycoprotein is a structural protein and is responsible for virus entry (attachment, membrane fusion, and virion assembly); it therefore represents a major target for neutralizing antibodies.

Table 2 shows the sets of primers used to target genetic sequences that code for the expression of the EBOV GP protein and the ZIKV E protein. EBOV and ZIKV were selected for this validation study because they were responsible for two of the most recent international epidemic emergencies of viral origin. The EBOV variant Makona was the causal agent of the West Africa Ebola epidemics [37,38], while ZIKV was the causal agent of a recent epidemic event that spanned most of Latin America and a significant portion of the United States in 2016 [5–10].

### Effect of amplicon length

**Fig 2:**
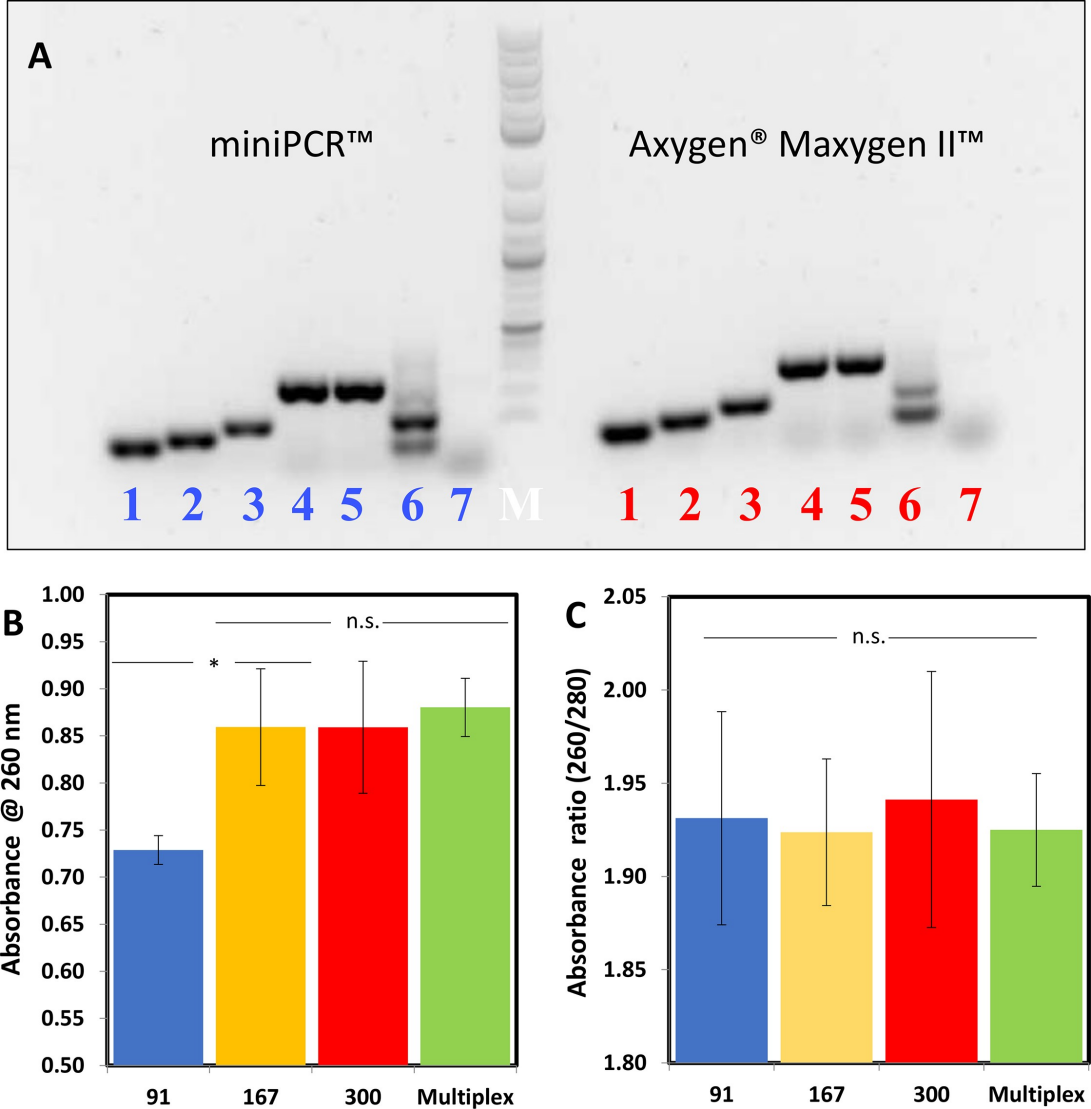
Comparison of the miniPCR and Maxygen thermocyclers using primer sets that yield different amplicon sizes. (A) Comparative images of agarose gel electrophoresis of DNA coding for EBOV GP protein using miniPCR and Maxygen thermocyclers. Different amplicon sizes obtained using the miniPCR (left section) and Maxygen (right section) thermal cyclers. From left to right: 91 bp (lane 1), 131 bp (lane 2), 167 bp (lane 3), 239 bp (lane 4), and 300 bp (lane 5), multiplex amplification of 91 and 167 bp (lane 6), and a negative control (lane 7). B) Evaluation of the amount of miniPCR-amplified DNA (as measured by Nanodrop Spectrometry at 260 nm), and (C) the purity (absorbance ratio at 260/280 nm) of DNA produced by primer sets that produce amplicons of different sizes. Primers that were aimed to produce amplicons of 91bp produced statistically lower amounts of DNA in miniPCR amplification experiments (p-value < 0.05). All primer sets yielded amplification products with statistically similar purities (p-value >> 0.05). Error bars indicate one standard deviation.

We conducted different sets of experiments to compare the performance of the miniPCR and a conventional commercial thermocycler. In a first round of experiments, we used five different sets of primers of different lengths that target different regions within the selected EBOV GP sequence. The lengths and sequences of each of the amplicons generated by each set of primers are presented in Tables 2 and 3. Fig 1C graphically shows the regions of the EBOV GP sequence to which each primer set binds and the length of each of the originated amplicons. These sets of primers generated PCR products of 91, 131, 167, 239, and 300 nucleotides (nt). Fig 2A shows images of a representative agarose gel experiment for the PCR products of different amplification reactions using different primer combinations. Products obtained using the miniPCR and the conventional thermocycler are presented. These results show that the use of primer sets designed to produce PCR products of different lengths has no meaningful effect on the performance of the amplifications. Moreover, we did not observe any substantial differences between the performance of the miniPCR and the conventional thermocycler. In addition, our use of a multiplex PCR strategy did not result in any obvious advantage over the use of conventional PCR; this observation holds for reactions conducted using both the miniPCR and the conventional thermocycler.

Interestingly, the profile of amplification products from the multiplexed reactions differed between the mini-PCR and the conventional thermocycler (Fig 2A). In an ideal multiplex PCR [39], where all targeted sequences are equally accessible to primer pairs and the polymerase, denser (more intense) bands are expected for the higher molecular weight amplification products (more base pairs per amplicon unit) than for the lower molecular weight products. Consistently, the higher molecular weight product of the multiplex reaction appears as a darker band in the miniPCR amplification. However, this is not the case for the conventional PCR results. This finding, which was consistent among experimental repeats, suggests a tighter control of the PCR microenvironmental conditions (i.e., mixing, temperature homogeneity, etc.) within the miniPCR reaction cells.

We further evaluated the effect of using primers that yield amplification products of different sizes (namely 91, 167, and 300 bp) in the miniPCR thermocycler. For this purpose, we conducted experiments in which we determined the amount of DNA produced by miniPCR amplifications using NanoDrop spectrophotometry. For this experimental set, we used 0.5 ng of EBOV DNA template. Our results suggest that the total amount of amplification product is statistically lower with the use of primers that flank a region of 91 bp (Fig 2B). This is an expected result; primers aimed at producing shorter amplicons should render less DNA per cycle. However, the purity of the DNA obtained in reactions using all different primer sets is statistically similar (Fig 2C). Moreover, the amount of amplification product is statistically similar for reaction protocols that yield amplicons of 167 and 300 bp (Fig 2B). These results suggest that the use of primers of about 150bp in size for miniPCR amplifications will render similar results, in terms of DNA quantity and quality, as those obtained with larger primers. The total amount and purity of amplification product was statistically similar for multiplex amplification (using primers that yield amplicons of 91 and 167 bp) and single primer amplification (using primers that yield amplicons of 167 or 300bp) (Fig 2B and 2C). This observation is based on final-point PCR results; in a real-time amplification, multiplexing might provide faster results.

### Analysis of sensitivity

**Fig 3:**
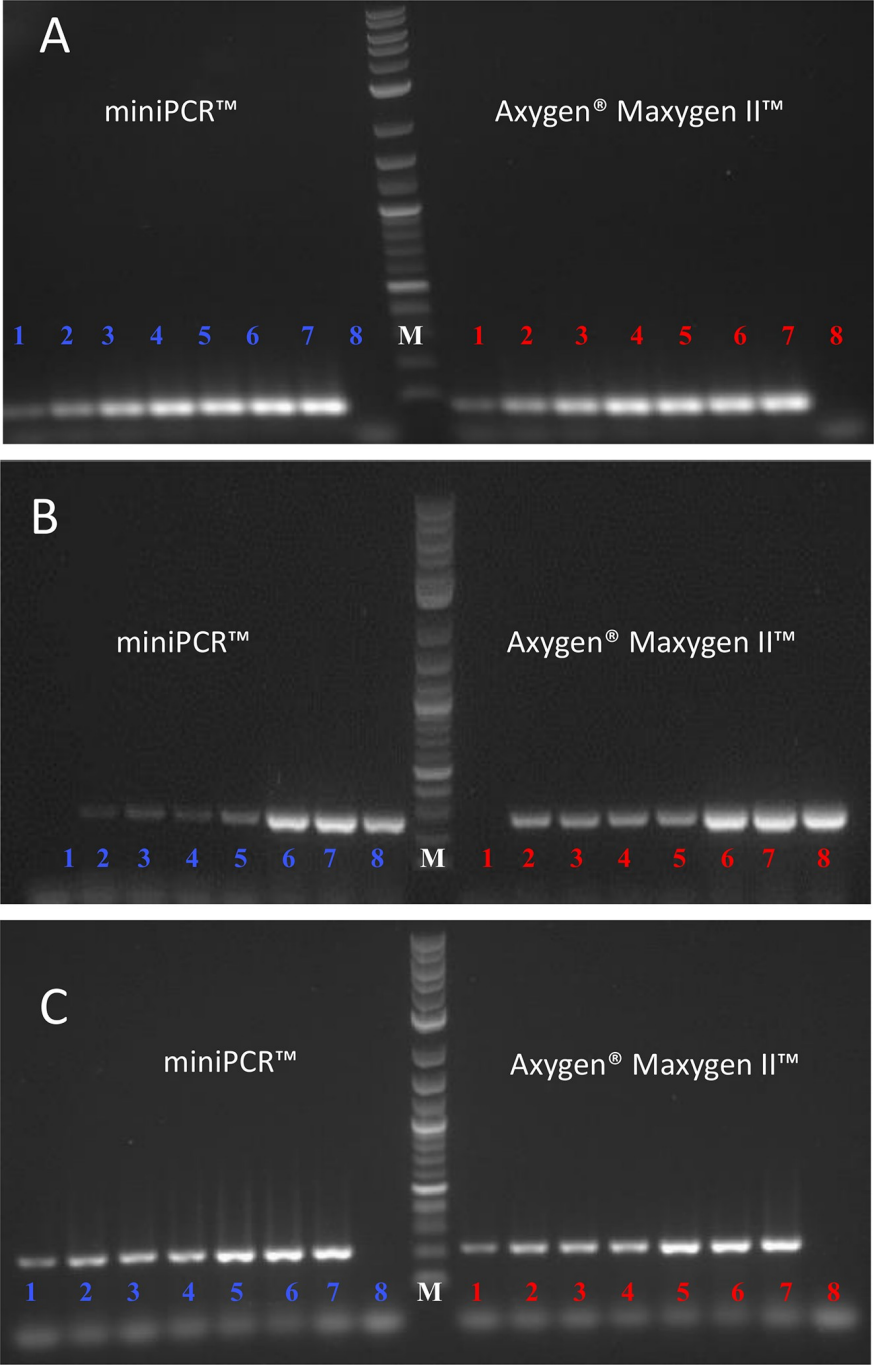
Comparison of sensitivity between the miniPCR and Maxygen thermal cyclers. A) Sensitivity trials using a 91 bp amplicon in miniPCR (left section) and Maxygen (right section) thermal cyclers. The initial template amount was gradually increased from left to right: 66 (lane 1), 6.62 x 10^2^ (lane 2), 6.62 x 10^4^ (lanes 3), 6.62 x 10^6^ (lane 4), 6.62 x 10^7^ (lane 5), 1.30 x 10^8^ (lane 6), and 3.9 x 10^8^ DNA copies (lane 7), and negative control (lane 8). B). Sensitivity trials using a 300bp amplicon in the miniPCR (left section) and Maxygen (right section) thermal cyclers. The initial template amount was gradually increased from left to right: negative control (lane 1), 66 (lane 2), 6.62 x 10^2^ (lane 3), 6.62 x 10^4^ (lanes 4), 6.62 x 10^6^ (lane 5), 6.62 x 10^7^ (lane 6), 1.30 x 10^8^ (lane 7), and 3.9 x 10^8^ DNA copies (lane 8). These results showed no significant differences between devices. (C) Sensitivity trials using a 252bp Zika virus amplicon in the miniPCR (left section) and Maxygen (right section) thermal cyclers. The initial template amount was gradually increased from left to right: 66 (lane 1), 6.62 x 10^2^ (lane 2), 6.62 x 10^4^ (lane 3), 6.62 x 10^6^ (lane 4), 6.62 x 10^7^ (lane 5), 1.30 x 10^8^ (lane 6), and 3.9 x 10^8^ DNA copies (lane 7), and negative control (lane 8).

Next, we conducted a second series of experiments to assess the sensitivity of the PCR reactions conducted in both thermocyclers using primers that generate amplicons of two different sizes. Fig 3A and 3B show the PCR products of amplification reactions conducted using a set of primers that produce amplicons of 91 nt and a 300 bp, respectively. In both cases, different concentrations of EBOV-GP genetic material, in the range of 5 × 10^−7^ to 3.0 ng/10 μL, were used as reaction template. The amplification proceeds with sufficient quality to allow proper visualization of the amplification product, even at low nucleic acid concentrations. However, we observed differences in the profile of the products of amplification when using primers to flank regions of 91 or 300 nt (compare Fig 3A and 3B). For instance, at low DNA template concentrations, the primers that flank the 91nt region appears to be more effective than those that target the 300nt region. In this set of experiments, we were particularly careful to ensure the integrity of the genetic material used as a template. Evidently, under real conditions, the integrity of larger templates can be more compromised by environmental factors when compared to shorter DNA segments, so targeting shorter nucleic DNA sequences might be advantageous [40,41]. Moreover, in the range of DNA concentrations explored, we did not observe any conclusive difference in performance between the miniPCR and the conventional thermocycler.

In an additional experiment, we challenged the sensitivity of both PCR thermocyclers with a different set of primers. This time, we amplified a ZIKV sequence, aiming to produce an amplicon of 252 base pairs (bp). Different concentrations of ZIKV genetic material, in the range of 5 × 10^−7^ to 3.0 ng/10 μL, were used as reaction template. Fig 2C presents an agarose gel showing the amplification products of this experiment. As before, we observed a similar performance between thermocyclers. Both the miniPCR and the conventional systems were able to generate a visible band of amplification products across the whole range of DNA dilutions.

### Robustness of the miniPCR system

**Fig 4:**
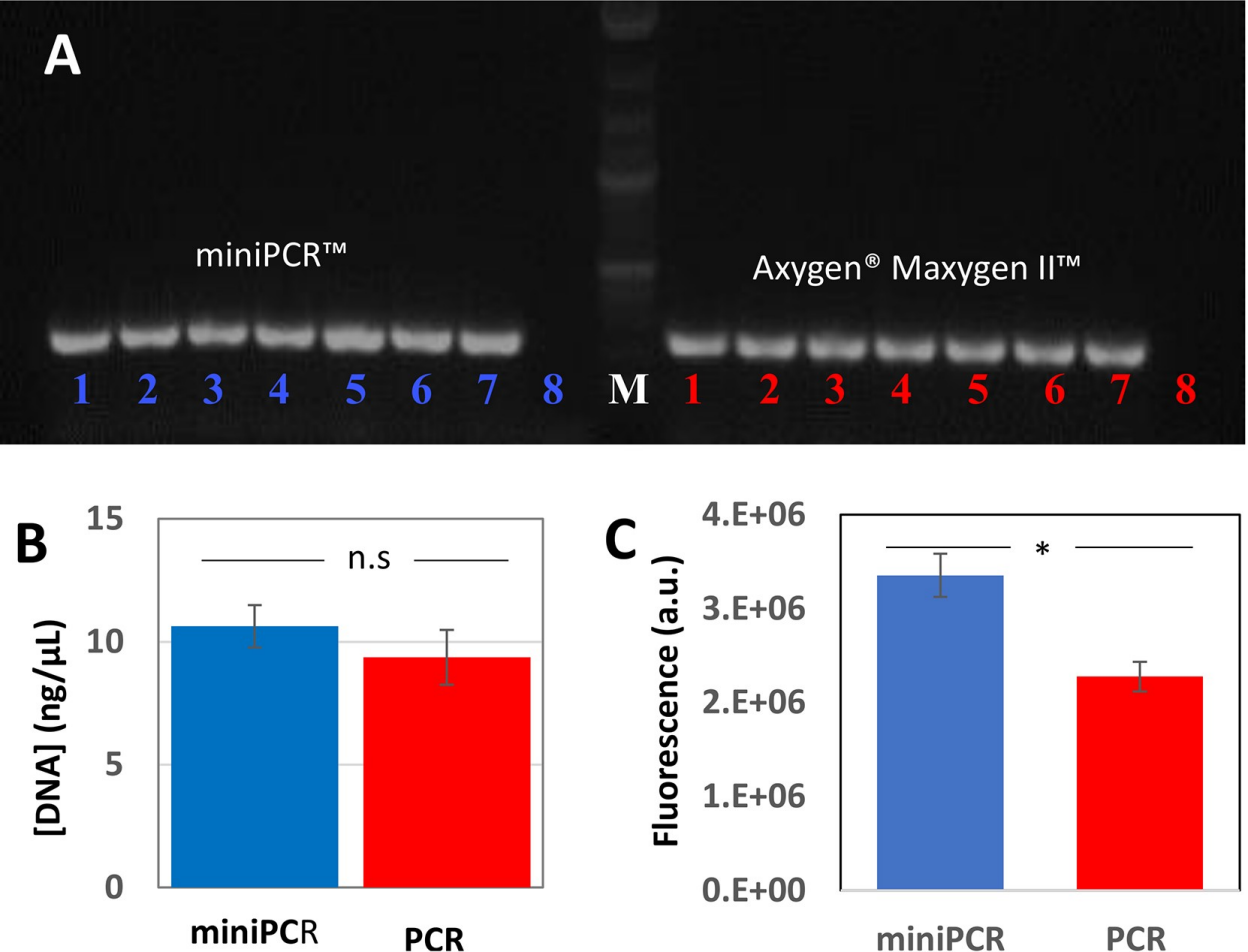
Comparison of the amount of amplification product produced by miniPCR (left section) and Maxygen (right section) thermal cyclers. A) Repeatability test using an initial amount of 0.5ng of a 300bp amplicon corresponding to the Ebola virus GP protein in the miniPCR (left section) and Maxygen (right section) thermal cyclers. Seven repeats (lane 1 to 7) and a negative control (lane 8) are shown. B) Average amount of nucleic acid material recovered from the agarose gels. No significant differences were noted between devices (p-value > 0.05). Error bars indicate one standard deviation. C) Average amount of nucleic acid material recovered, as quantified by SYBR Green qPCR staining and fluorescence measurements in a real time thermocycler. Significant differences were noted between devices (p-value* = 0.043). Error bars indicate one standard deviation.

In a final set of experiments, we tested the robustness of the miniPCR system from two different angles. First, we validated the reproducibility of the results generated by the miniPCR between different assay wells. For that purpose, we dispensed the same sample of DNA (EBOV genetic material; at 0.5 ng/μL; expected amplicon of 300bp) in seven of the eight amplification wells of the miniPCR thermocycler (one well was left as a negative control). For comparison, the same amplification experiment was performed in eight of the amplification cells of the conventional PCR system, selected at random. Fig 4A shows an agarose gel of the amplification products for eight different wells of both thermocyclers. We did not observe any significant variations in intensity among the wells in any thermocycles. In addition, we quantified the intensities of the bands corresponding to amplification products using band purification and nanodrop quantification. The results revealed small standard deviations for both sets of samples. Both thermocyclers also performed similarly in terms of reproducibility; the average amounts of amplification products were statistically similar for both data sets (α-value = 0.5; Fig 4B).

In an additional set of experiments, we quantified the amount of DNA amplified in different assay wells (in both thermocyclers) using SYBR Green staining followed by fluorescence determination in a RT-PCR apparatus (Fig 4C). The results confirm that both thermocyclers are similar in terms of reproducibility, as both exhibited a variance coefficient of ~0.06. Interestingly, in these experiments (without purification), the miniPCR thermocycler produced significantly higher amounts of DNA than were obtained with the conventional thermocycler.

**Fig 5:**
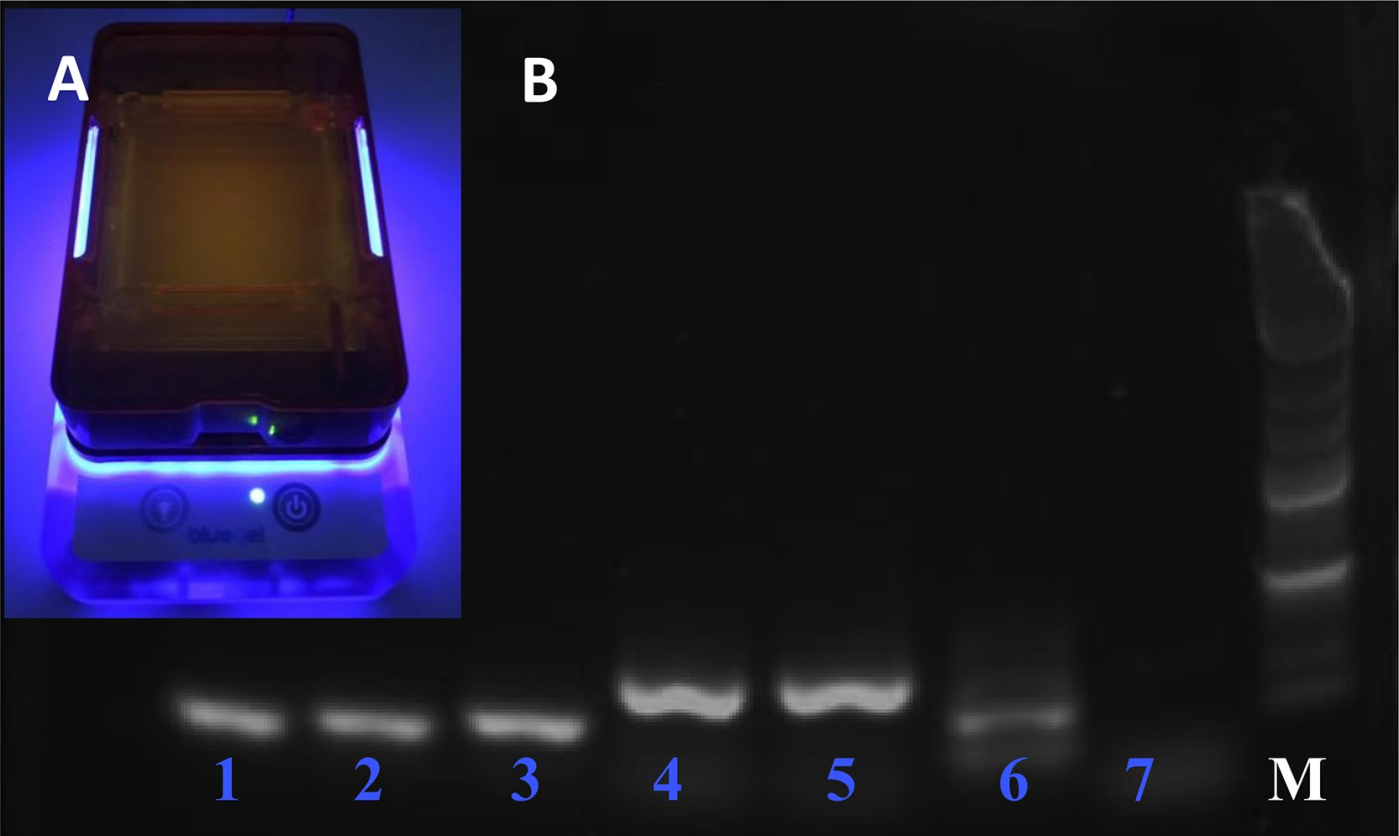
The blueGel electrophoresis chamber: blueGel (A) allows visualization of a 15 ml agarose gel using an integrated blue LED array. B) Agarose gel electrophoresis of Ebola virus amplification products of different sizes using the miniPCR thermocycler and blueGel electrophoretic chamber: 91 bp (lane 1), 131 bp (lane 2), 167 bp (lane 3), 239 bp (lane 4), 300 bp (lane 5), multiplex amplification of 91and 167 bp (lane 6), and negative control (lane 7).

In all previously described experiments, the products of amplification from both thermocyclers were primarily detected on agarose gels using conventional electrophoresis techniques conducted with conventional lab equipment. However, as previously mentioned, the miniPCR system is commercialized with its own “blueGel” electrophoretic unit (Fig 5A). In a last set of experiments, we separated the amplification products using the miniPCR electrophoretic unit to assess its practicality and reliability. Fig 5B shows a gel that displays the products of amplification of a typical experiment where EBOV sequences were targeted.

Besides its portability, the blueGel electrophoretic unit has other important advantages: its size allows optimization of reagent usage (agarose gel 15ml, buffer 25ml), the built-in power supply allows visualization of band separation in real time, which can shorten the electrophoretic time by up to 5 minutes; and exposure to ethidium bromide and UV light is completely avoided by the use of GelGreen dye and detection with blue light.

## Conclusions

The challenge of POC detection of viral threats is of paramount importance, particularly in underdeveloped regions and in emergency situations (i.e., natural disasters or epidemic outbreaks). In the context of an emergency, time is very limited (as are other resources) to do research or develop new technologies; therefore, the use of commercially available and tested technologies is an obvious first countermeasure. Our research extends the validation of the miniPCR technology to the as yet unexplored topic of detection of Ebola and Zika, and we have validated six sets of primers for Ebola and one set for Zika detection. Our results suggest that the capacity of selective amplification in a conventional thermocycler and in a miniPCR is essentially the same. The use of the miniPCR is intuitive and simple; the user can easily follow the advance of the iterative temperature cycling using a laptop. Despite its compact size, the miniPCR allows a full amplification protocol to be performed in a similar time as in a conventional thermocycler (a difference of only six minutes in a 30-cycle protocol). The mini-PCR thermocycler exhibits the essential attributes of a POC system: (a) the use of small volumes, (b) low capital cost, (c) portability, (d) and a fast, accurate, and selective response. Therefore, this already commercially available and simple nucleic acid amplification system has great potential for use in remote or underprivileged areas, in the case of natural disasters, on the battlefield, or during epidemic emergencies.

In addition, we have documented the effect of using different primers that yield different amplicon sizes, thereby providing a reference resource for primer design for POC applications using the miniPCR. We also provided a detailed account of a strategy for producing Ebola- and ZIKV-related nucleic acids that is free of infection risk in any conventional lab. This is a useful, if not necessarily intuitive, resource that allows personnel to study the virus nucleic acids without the risk of contracting a deadly disease. This straightforward approach will facilitate POC research related to highly infective viruses in underdeveloped regions.

## Supporting information

S1 Dataset Dataset related to Fig 2B and 2C. Evaluation of the amount of miniPCR-amplified DNA (as measured by Nanodrop Spectrometry at 260 nm), and the purity (absorbance ratio at 260/280 nm) of DNA produced by primer sets that produce amplicons of different sizes.
(XLSX)

S2 Dataset Dataset related to Fig 4B. Average amount of nucleic acid material recovered from the agarose gels.
(XLSX)

S3 Dataset Dataset related to Fig 4C. Average amount of nucleic acid material recovered, as quantified by SYBR Green qPCR staining and fluorescence measurements in a real time thermocycler.
(XLSX)

S1 Fig Maps and sequences related to EBOV. A)Vector Map: pCMV Ebola GP. Mammalian Expression Vectors, ampicilin and neomycin resistance, CMV promoter and primers EBOV1, EBOV2, EBOV3, EBOV4, EBOV5 and EBOV6. B) Amino acid sequence alignment from EBOV GenBank 1976–2014 (GP Protein).
(PDF)

S2 Fig Maps and sequences related to ZIKV. A)Vector map: PUC57 Zika E Clonning vector, ampicilin resistance and primers ZIKV1 y ZIKV2. B) Amino acid sequence alignment from ZIKV GenBank 2013–2016 (E Protein).
(PDF)
