# Supplementary material for: Validation of use of the miniPCR thermocycler for Ebola and Zika virus detection
Source: PLoS One. 2019 May 9;14(5):e0215642. doi: 10.1371/journal.pone.0215642 (PMC6508694; doi:10.1371/journal.pone.0215642)
Supplement: S2 Fig — A)Vector map: PUC57 Zika E Clonning vector, ampicilin resistance and primers ZIKV1 y ZIKV2. B) Amino acid sequence alignment from ZIKV GenBank 2013–2016 (E Protein). (PDF) [file pone.0215642.s005.pdf]

A

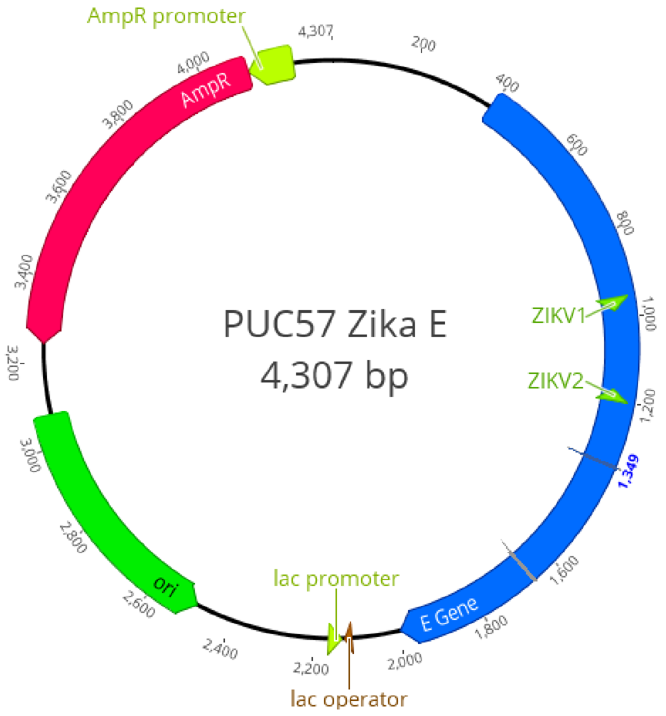

A)Vector map: PUC57 Zika E Clonning vector, ampicilin resistance and primers ZIKV1 y ZIKV2

B

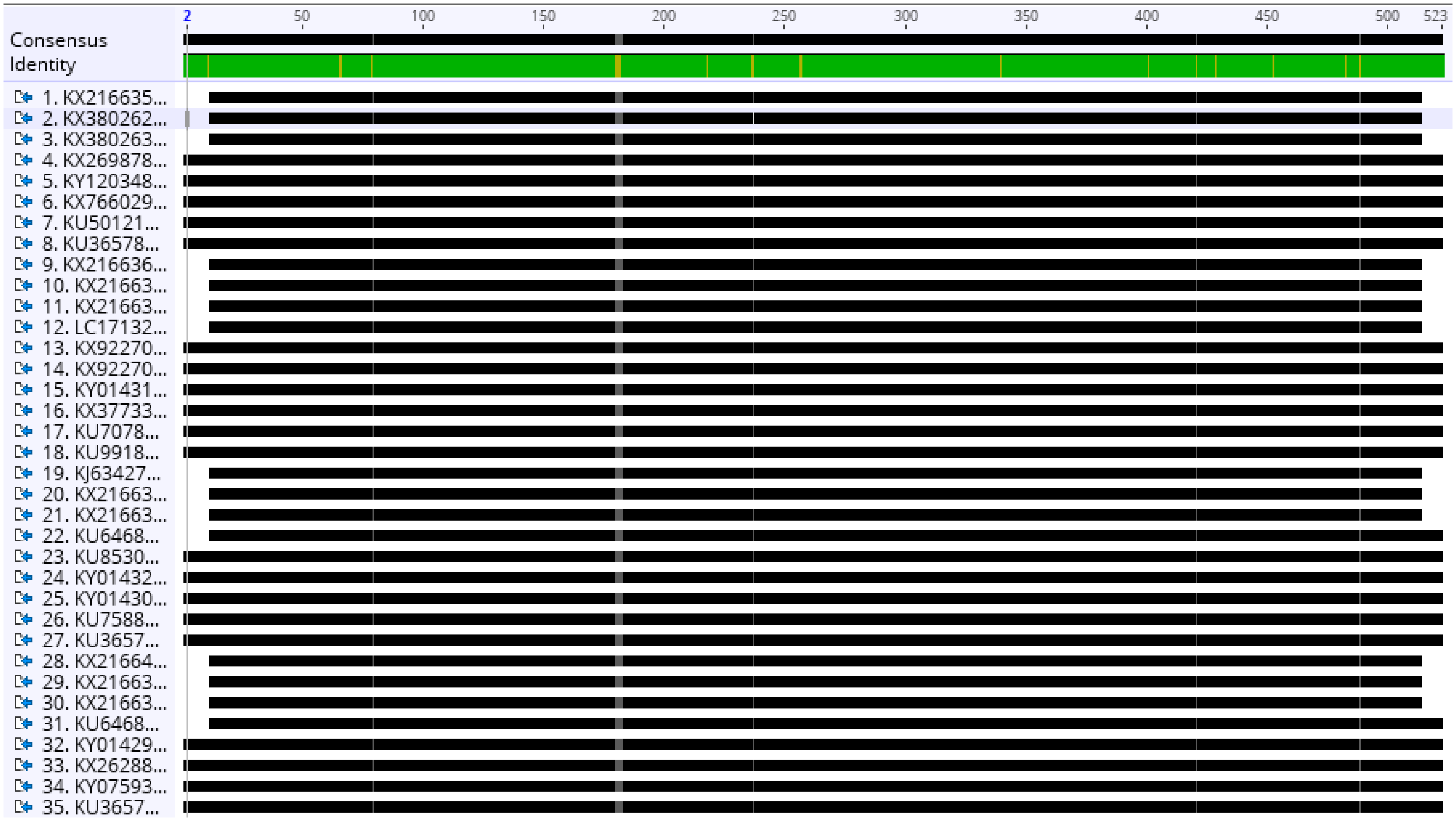

B) Amino acid sequence alignment from ZIKV GenBank 2013–2016 (E Protein)
