# Supplementary material for: Validation of use of the miniPCR thermocycler for Ebola and Zika virus detection
Source: PLoS One. 2019 May 9;14(5):e0215642. doi: 10.1371/journal.pone.0215642 (PMC6508694; doi:10.1371/journal.pone.0215642)
Supplement: S1 Fig — A)Vector Map: pCMV Ebola GP. Mammalian Expression Vectors, ampicilin and neomycin resistance, CMV promoter and primers EBOV1, EBOV2, EBOV3, EBOV4, EBOV5 and EBOV6. B) Amino acid sequence alignment from EBOV GenBank 1976–2014 (GP Protein). (PDF) [file pone.0215642.s004.pdf]

A

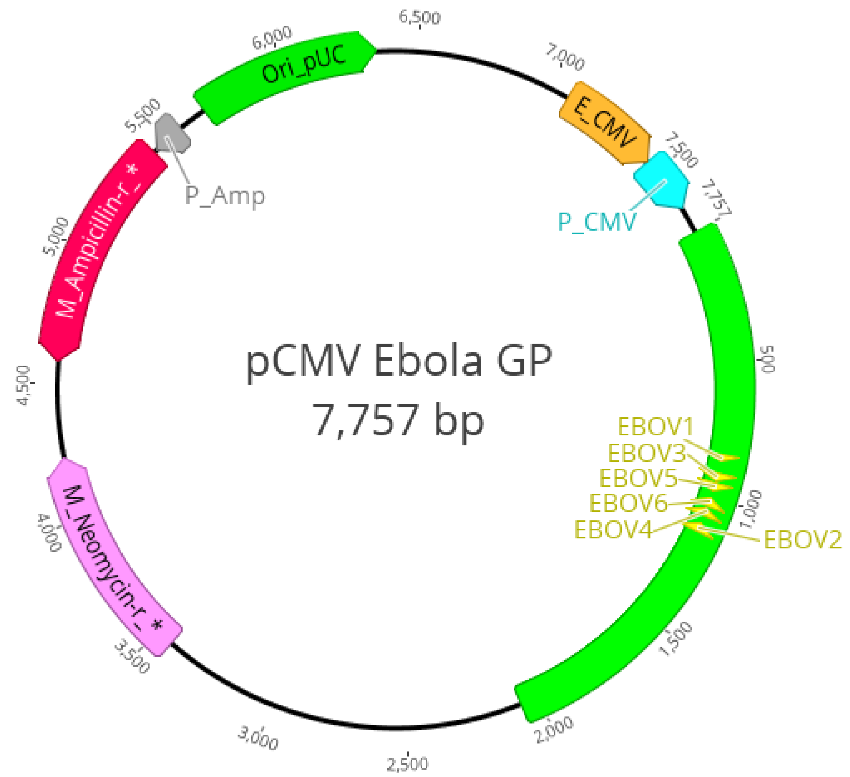

A)Vector Map: pCMV Ebola GP. Mammalian Expression Vectors, ampicilin and neomycin resistance, CMV promoter and primers EBOV1, EBOV2, EBOV3, EBOV4, EBOV5 and EBOV6

B

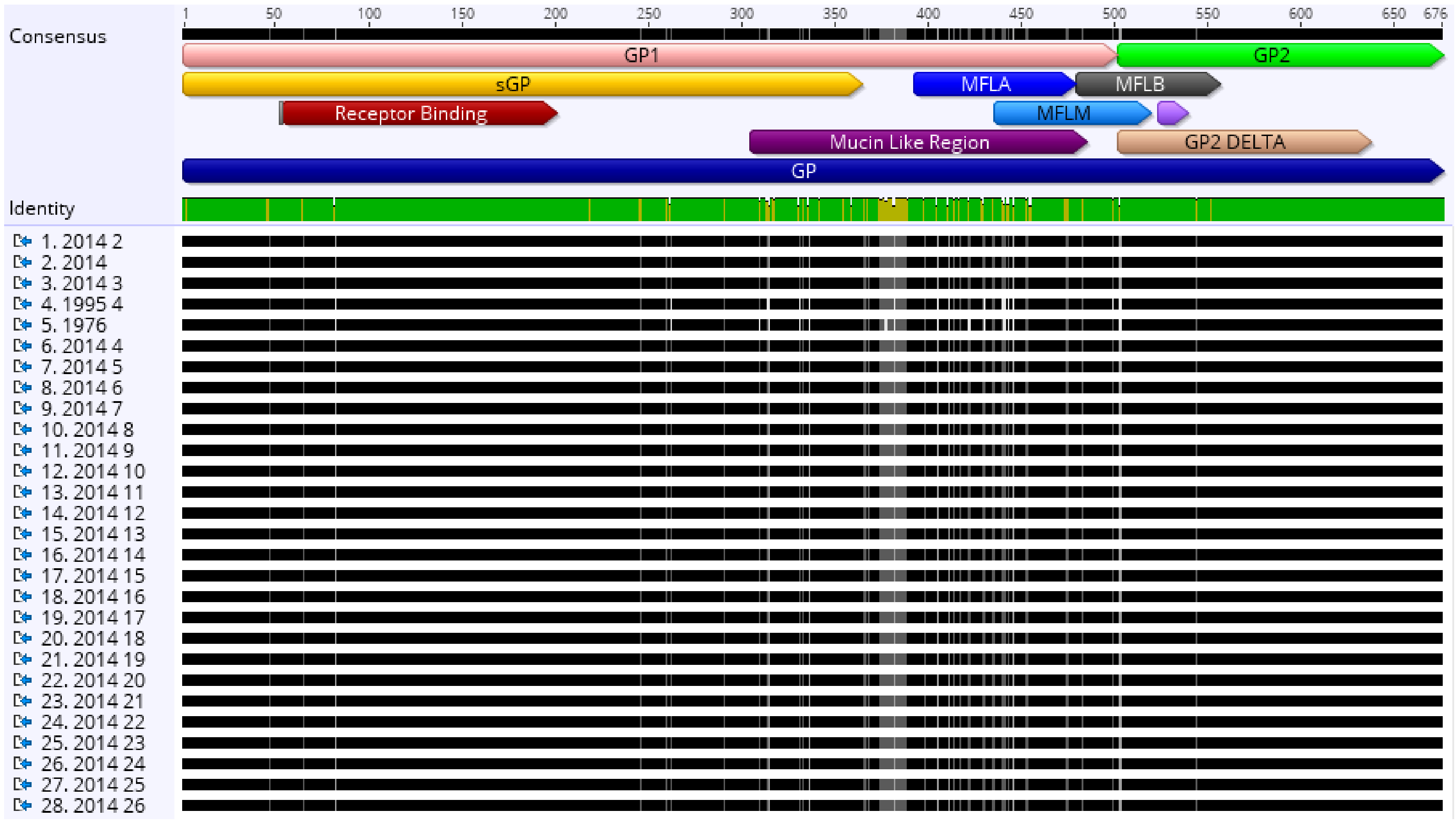

B) Amino acid sequence alignment from EBOV GenBank 1976–2014 (GP Protein)
